# Characteristics and outcomes of neonates with intrapartum asphyxia managed with therapeutic hypothermia in a public tertiary hospital in South Africa

**DOI:** 10.1186/s12887-023-03852-2

**Published:** 2023-01-31

**Authors:** Firdose Lambey Nakwa, Letlhogonolo Sepeng, Alison van Kwawegen, Reenu Thomas, Karabo Seake, Tshiamo Mogajane, Nandi Ntuli, Claude Ondongo-Ezhet, Samantha Kesting, Dikeledi Maureen Kgwadi, Noela Holo Bertha Kamanga, Annaleen Coetser, Jeanne Van Rensburg, Michael S. Pepper, Sithembiso C. Velaphi

**Affiliations:** 1 Department of Paediatrics and Child Health, School of Clinical Medicine, Faculty of Health Sciences, Chris Hani Baragwanath Academic Hospital, University of the Witwatersrand, Soweto, Johannesburg South Africa; 2 Department of Immunology, SAMRC Extramural Unit for Stem Cell Research and Therapy, Faculty of Health Sciences, Institute for Cellular and Molecular Medicine, University of Pretoria, Pretoria, South Africa

**Keywords:** Neonates, Intrapartum asphyxia, Therapeutic hypothermia, Low- and Middle- Income Countries (LMIC), Mortality, Hypoxia Ischaemic Encephalopathy (HIE)

## Abstract

**Background:**

In randomized clinical trials, therapeutic hypothermia (TH) has been shown to reduce death and/or moderate-to-severe disability in neonates with hypoxic ischemic encephalopathy (HIE) in high-income countries, while this has not consistently been the case in low-and middle-income countries (LMICs). Many studies reporting on outcomes of neonates with HIE managed with TH are those conducted under controlled study conditions, and few reporting in settings where this intervention is offered as part of standard of care, especially from LMICs. In this study we report on short-term outcomes of neonates with moderate-to-severe HIE where TH was offered as part of standard of care.

**Objective:**

To determine characteristics and mortality rate at hospital discharge in neonates with moderate-to-severe HIE.

**Methods:**

Hospital records of neonates with intrapartum asphyxia were reviewed for clinical findings, management with TH (cooled or non-cooled) and mortality at hospital discharge. Inclusion criteria were birthweight ≥ 1800 g, gestational age ≥ 36 weeks and moderate-to-severe HIE. Comparisons were made between survivors and non–survivors in cooled and/or non-cooled neonates.

**Results:**

Intrapartum asphyxia was diagnosed in 856 neonates, with three having no recorded HIE status; 30% (258/853) had mild HIE, and 595/853 (69%) with moderate-to-severe HIE. The overall incidence of intrapartum asphyxia was 8.8/1000 live births. Of the 595 with moderate-to-severe HIE, three had no records on cooling and 67% (399/592) were cooled. Amongst 193 non-cooled neonates, 126 (67%) had documented reasons for not being cooled with common reasons being a moribund neonate (54.0%), equipment unavailability (11.1%), pulmonary hypertension (9.5%), postnatal age > 6 h on admission (8.7%), and improvement in severity of encephalopathy (8.7%). Overall mortality was 29.0%, being 17.0% and 53.4% in cooled and non-cooled infants respectively. On multivariate analysis, the only factor associated with mortality was severe encephalopathy.

**Conclusion:**

Overall mortality in neonates with moderate-to-severe HIE was 29.0% and 17.0% in those who were cooled. Cooling was not offered to all neonates mainly because of severe clinical illness, equipment unavailability and delayed presentation, making it difficult to assess overall impact of this intervention. Prospective clinical studies need to be conducted in LMIC to further assess effect of TH in short and long-term outcomes.

## Introduction

Intrapartum asphyxia has a higher incidence in low- and middle-income countries (LMIC) with an incidence of 20 per 1000 compared to 1–3 per 1000 live births in high-income countries (HIC) [1]. Intrapartum asphyxia accounts for 1 million deaths annually and is associated with high morbidity, common one being hypoxic ischemic encephalopathy (HIE) [2–4]. Neonates with HIE are classified as mild, moderate and severe according to the Sarnat classification [5]. There are multitudes of clinical trials that have investigated interventions to prevent death and/or disability in neonates with intrapartum asphyxia. Therapeutic hypothermia (TH) is one of the management strategies that has been shown to reduce death and/or moderate to severe disability at 18–24 months in neonates with moderate to severe HIE [6–9].

A Cochrane review in 2013 concluded that TH was associated with a 26% reduction in mortality with a number needed to treat of 11 [10]. A meta-analysis, looking at studies from both HIC and LMIC reported that TH is associated with a reduction in mortality more so in LMICs [11]. This reduction is seen for both methods of cooling (head and whole body cooling). Recently, a multicenter randomized clinical trial (HELIX trial) from LMICs reported that TH was associated with increased mortality, resulting in authors of this paper calling for an immediate cessation of cooling in LMICs [12]. TH continues to be used as an intervention to reduce the incidence of abnormal neurodevelopmental outcomes in neonates with intrapartum asphyxia in many centers in LMIC [13–16]. With concerns raised by the HELIX trial, it is important that continued use of TH in LMIC be monitored with a special focus on mortality. Matthew et al. concluded that effects of TH on mortality are uncertain but that one cannot deny the protective effect on neurodevelopmental outcomes seen in survivors and recommend that further studies be conducted in diverse settings [17].

There are limited studies reporting on the effect of TH in sub-Saharan Africa. A small randomized clinical trial from Uganda reported a high mortality in neonates managed with TH compared to those receiving standard care (33% vs 6.7%) [18]. A number of observational studies from South Africa where TH was implemented as part of standard care, reported mortality rates of 13–20% [15, 16, 19–21]. Many of these South African studies reporting these relative lower mortality rates were from relative small cohorts of neonates with HIE. Routine use of TH in LMIC needs to be monitored as the impact of TH on mortality, based on randomized clinical trials is questionable [12, 18]. In this study, we sought to assess characteristics and mortality rate at hospital discharge in a large cohort of neonates with moderate to severe encephalopathy from a large center in a LMIC where TH is used as part of standard of care.

## Methods

### Study design

A retrospective, descriptive analytic study of neonates diagnosed with moderate to severe HIE.

### Study setting

The study was conducted at Chris Hani Baragwanath Academic Hospital (CHBAH), Johannesburg, South Africa. CHBAH is situated in the southern part of Gauteng province, and serves the southwestern townships (Soweto) of Johannesburg with a population of about 1.3 million. It is a referral center for all neonates requiring admission from local clinics and for referrals from the only district hospital in Soweto. CHBAH conducts about 20 000 births per annum and the local clinics, and district hospital conduct about 10 000 and 3 000 births per annum respectively. All neonates diagnosed with moderate to severe encephalopathy born at CHBAH or referred from local clinics and hospitals are offered TH, using the TOBY criteria [22]. Neonates considered for TH include those born at 1) ≥ 36 weeks gestational age, a birthweight ≥ 1800 g and < 6 h of age at initiation of TH; 2) any one of the following: < 7.00 or base deficit ≥ 16 mmol/L based on an arterial or venous blood gas done within 60 min of birth; Apgar score ≤ 5 at 10 min after birth; continued need for resuscitation (including endotracheal and/or bag mask ventilation) for ≥ 10 min; 3) moderate or severe encephalopathy on clinical examination (Thompson score > 10) and 4) an abnormal amplitude-integrated electroencephalography (aEEG) of at least 30 min duration. The abnormalities of the aEEG could be any of the following: moderate abnormal background (upper margin of the band above 10 µV and lower margin below 5 µV), severe abnormal background (upper margin of the band below 10 µV and lower margin below 5 µV); normal background with seizure activity. If an aEEG is not available but the neonate meets the other three criteria, TH should still be considered. TH would be contraindicated to neonates with the following conditions: 1) neonates with no spontaneous respiratory effort after 30 min post-resuscitation (after excluding possible effect of anaesthesia or medication) or 2) a heart rate below 100 per minute at 20 min post-resuscitation, 3) neonates older than 6 h of age at the time of starting cooling and 4) those with major congenital abnormalities or signs suggestive of a syndrome or chromosomal abnormalities that involve brain dysgenesis.

In the earlier years of cooling a Thompson score of ≥7 was used to diagnose encephalopathy requiring TH, however we noted that neonates with mild HIE were being offered TH, thus the criteria was revised in 2018 and changed the Thompson score to qualify for cooling to that of ≥10. Neonates considered moribund included those who were expected to have immediate demise or had not initiated spontaneous breathing or only having gasping breathing by 30 minutes of resuscitation even if they had a heart rate.

### Study population

Infants who were diagnosed with intrapartum asphyxia, weighing ≥1800 grams with a gestation age of ≥36 weeks and were assessed to have moderate to severe encephalopathy, therefore qualified for TH, and had information on whether they were cooled or not were included in the study.

### Study procedures and data collection

Hospital records of newborn infants who were diagnosed with asphyxia were retrieved and the following data were collected: birth weight, sex, gestational age, Apgar score, time to onset of spontaneous respiration after birth, blood gas parameters from radial arterial blood taken within the first hour of birth, diagnosis of HIE, and survival to hospital discharge.

### Data analysis

Means with standard deviations, medians with ranges were used to summarize continuous variables while frequencies and percentages were used to describe categorical variables. The Chi square with Yates correction or Fischer’s exact tests and Student t-test were used to compare the categorical variables and continuous variables respectively between non-survivors and survivors. Differences with *p*-values <0.05 were considered statistically significant. The extent of association of different variables with severe outcome were reported using odds ratio and the precision using the 95% confidence interval. In order to determine predictors of mortality variables with *p*-values <0.1 on univariate analysis were included in the multivariate logistic regression model. All variables were considered equally for the univariate analysis. STATA version 15.0 was used to perform the statistical analysis. Approval to conduct the study was sought and obtained from the hospital protocol review committee and the University of the Witwatersrand Human Research Ethics Committee (Protocol M151100).

## Results

**Fig. 1:**
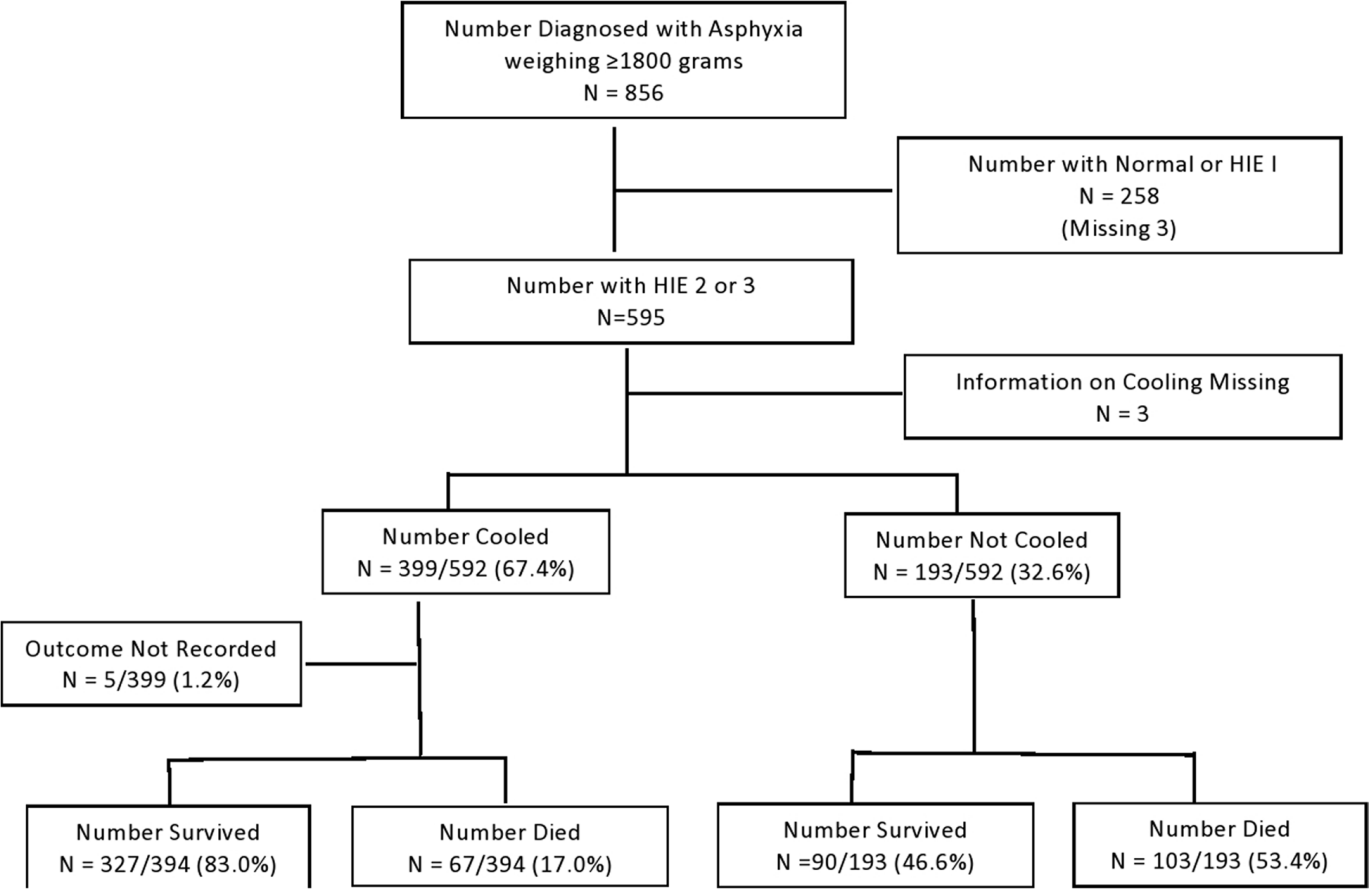
Flow diagram of neonates included in the study

There were 856 neonates weighing ≥ 1 800 g diagnosed with intrapartum asphyxia from 2015 to 2019 from 96 784 live births, giving an incidence of asphyxia at 8.8/1000 live births and 5.5/1000 when all live births (155993) for the cluster are considered. Two hundred and fifty-eight (30.1%) were assessed to have normal neurological examination or mild HIE, and three (0.4%) did not have records regarding their neurological examination, thus a total of 595 neonates had moderate to severe encephalopathy. Four hundred and forty-six (52.3%) were diagnosed with moderate and 149/853 (17.5%) with severe encephalopathy. The incidence of moderate and severe encephalopathy was 4.6/1000 and 1.5/1000 live births respectively. Amongst the 595 with moderate to severe encephalopathy, three did not have information regarding whether they were cooled or not and thus, 592 were considered for further analysis. Of the 592 patients with information on cooling, 399 (67%) were cooled (Fig. 1).

Over the 5-year study period, the proportion of neonates that were cooled amongst those with moderate to severe encephalopathy ranged from 61.2 to 74.6%, with the first two years having the highest proportion of cooled neonates (Table 1). Amongst the 193 neonates who were not cooled, reasons for not cooling them were not documented in 67 (34.7%). Amongst the 126 with known reasons for not cooling, the common reasons were that the neonate was moribund (54.0%), unavailability of equipment (cooling machine) (11.1%), diagnosis of persistent pulmonary hypertension (9.5%), late presentation (> 6 h postnatal age at arrival or diagnosis (8.7%), improvement in HIE severity (8.7%) and gestational age < 36 weeks (7.2%).

**Table 1:** Proportion of neonates with moderate to severe asphyxia managed with therapeutic hypothermia over a 5-year period

Year	Number of Neonates with Moderate to Severe Asphyxia	Proportion Managed with Therapeutic Hypothermia n (%)
2015	114	85 (74.6)
2016	159	111 (69.8)
2017	133	88 (66.2)
2018	98	60 (61.2)
2019	88	55 (62.5)
Total	592	399 (67.4%)

In comparing neonates who were cooled to those who were not cooled, those who were not cooled were more likely to be of lower birth weight (3 058 vs 2 961 g, *p* < 0.001), lower gestational age (38.1 vs 38.7 weeks, *p* < 0.001), had lower 5-min Apgar scores (3 vs 5, *p* < 0.001), took longer to achieve spontaneous respiration (14 vs 7 min, *p* < 0.001), had a lower pH (6.90 vs 7.05, *p* < 0.001), and had a higher Thompson score (14 vs 11, *p* < 0.001) (Table 2). Proportionately, there were also more with severe encephalopathy in the non-cooled compared to the cooled group (56.5 vs 10%, *p* < 0.001).

**Table 2:** Univariate analysis comparing characteristics of neonates with moderate to severe encephalopathy between cooled and not cooled groups

	Cooled *n* = 399	Not Cooled *n* = 193	*p*-value
Birth weight categories			< 0.001
≥ 2500 g	348/399 (87.2)	148/193 (76.7)	
< 2500 g	51/399 (12.8)	45/193 (23.3)	
Mean birth weight, in grams	3058 ± 508	2961 ± 565	0.036
Gestational age categories			0.003
≥ 38 weeks	312/397 (78.6)	127/189 (67.2)	
< 38 weeks	85/397 (21.4)	62/189 (32.8)	
Mean gestational age, in weeks	38.1 ± 2.5	38.7 ± 2.2	0.006
Sex, male	247/397 (62.2)	118/193 (61.1)	0.801
Apgar score at 5 min			< 0.001
≥ 4	331/392 (84.4)	88/183 (48.1)	
0–3	61/392 (15.6)	95/183 (51.9)	
Median Apgar score at 5 min	5 (4–6)	3 (2–6)	< 0.001
Time to First Breath			< 0.002
< 20 min	257/303 (84.8)	67/110 (60.9)	
≥ 20 min	46/303 (15.2)	43/110 (39.1)	
Median	7 (3–13)	14 (5–40)	< 0.001
Sarnat staging			< 0.001
Stage 2 (moderate encephalopathy)	359 (90.0)	84 (43.5)	
Stage 3 (severe encephalopathy)	40 (10.0)	109 (56.5)	
Thompson score			< 0.001
≤ 10	185/384 (48.2)	43/148 (29.0)	
> 10	199/384 (51.8)	105/148 (71.0)	
Median Thompson score	11 (9–13)	14 (10–16)	< 0.001
Blood gas, pH			< 0.001
≥ 7.00	253/398 (63.6)	63/179 (32.2)	
< 7.00	145/398 (36.4)	116/179 (64.8)	
Median pH	7.05 (6.90–7.16)	6.90 (6.70–7.10)	< 0.001
Blood gas, Base deficit (mmols/L)			0.173
≤ 16.0	104/391 (26.6)	48/177 (27.1)	
> 16.0	287/391 (73.4)	129/177 (72.9)	
Median base deficit	19.9 (16.0–24.0)	22.3 (16.0–27.1)	0.001

Five neonates (1.2%) in the cooled group had missing information on outcome at discharge. Amongst those with known outcome overall, 170/587 (29.0%) neonates diagnosed with moderate to severe encephalopathy had died at hospital discharge. There were 67/394 (17.0%) deaths in the cooled and 103/193 (53.4%) deaths in the non-cooled neonates. This difference was found to be significant upon Chi-squared analysis following Yates correction (*p* < 0.05). The mortality for those who were cooled between the years 2015 – 2017, was 17.9% (51/284); 17.5% (44/251) had Thomson scores ≥ 7. In 2018 -2019, 13.9% (16/115) of infants who were cooled died; of these 14.8% (13/88) had a Thomson scores ≥ 10.

On univariate analysis factors associated with mortality in the cooled neonates included gestational age < 38 weeks (OR 1.82, 95% CI 1.00–3.31), time to first breath > 20 min (OR 2.64, 95% CI 1.25 -5.58), severe encephalopathy (OR 5.77, 95% CI 2.81–11.8), and a Thompson score > 10 (OR- 2.11, 95% CI 1.20–3.71). None of these factors were noted to be significantly associated with mortality on multivariate analysis (Table 3).

**Table 3:** Factors associated with mortality amongst neonates with moderate to severe encephalopathy who were cooled

	Non-survivors *n* = 67	Survivors *n* = 327	Univariate analysis		Multivariate analysis	
			OR (95% CI)	*p*-value	aOR (95% CI)	*p*-value
Birth weight categories					N/A	
≥ 2500 g	56 (83.6)	291 (89.0)	1	_		
< 2500 g	11 (16.4)	36 (11.0)	0.63 (0.30–1.31)	0.213		
Gestational age categories						
≥ 38 weeks	46 (69.7)	217 (66.4)	1	_	1	_
< 38 weeks	20 (30.3)	63 (19.3)	1.82 (1.00–3.31)	0.045	1.47 (0.70–3.11)	0.307
Sex					N/A	
Female	27 (40.3)	122 (37.5)	1	_		
Male	40 (59.7)	203 (62.5)	0.89 (0.52–1.52)	0.672		
Apgar score at 5 min					N/A	
≥ 4	52/66 (78.8)	275/321 (85.7)	1	_		
0–3	14/66 (21.2)	46/321 (14.3)	1.61 (0.82–3.15)	0.16		
Time to First Breath						
< 20 min	35/48 (72.9)	220/251 (87.6)	1	_	1	_
≥ 20 min	13/48 (27.1)	31/251 (12.4)	2.64 (1.25–5.58)	0.008	1.86 (0.74–4.63)	0.184
Sarnat staging						
Stage 2 (moderate encephalopathy)	48/67 (71.6)	306/327 (93.6)	1	_	1	_
Stage 3 (severe encephalopathy)	19/67 (28.4)	21/327 (6.42)	5.77 (2.81–11.8)	0.001	2.04 (0.74–5.67	0.17
Thompson score						
≤ 10	22/65 (33.8)	163/314 (51.9)	1	_	1	
> 10	43/65 (66.2)	151/314 (48.1)	2.11 (1.20–3.71)	0.008	1.52 (0.75–3.08)	0.248
Blood gas, pH						
≥ 7.00	36/67 (53.7)	214/326 (65.6)	1	_	1	
< 7.00	31/67 (46.3)	112/326 (34.4)	1.65 (0.96–2.81)	0.065	1.01 (0.51–2.00)	0.981
Blood gas, base deficit (mmols/L)					N/A	
≤ 16	17/66 (25.8)	85/320 (26.6)	1	_		
> 16	49/66 (74.2)	235/320 (73.4)	1.04 (0.57–1.91)	0.893		

Amongst the non-cooled neonates, factors associated with mortality on univariate analysis were 5-min Apgar score < 3 (OR 11.2, 95% CI 4.94- 25.6), time to first breath > 20 min (OR 6.65, 95% CI 2.77–16.0), severe encephalopathy (OR 25.9, 95% 9.17–73.0), a Thompson score > 10 (OR 19.2, 95% CI 4.69–78.8), a pH < 7.00 (OR 7.57, 95% CI 3.18–18.0), and a high base deficit (OR 4.90, 95% CI 2.22–10.8). On multivariate analysis, the only factor that was associated with mortality was severe encephalopathy (OR 51.5, 95% CI 5.86–453; Table 4).

**Table 4:** Factors associated with mortality amongst neonates with moderate to severe encephalopathy who were not cooled

	Non-survivors *n* = 103	Survivors *n* = 90	Univariate analysis		Multivariate analysis	
			OR (95% CI)	*p*-value	OR (95% CI)	*p*-value
Birth weight categories						
≥ 2500 g	76/103 (73.8)	72/90 (80.0)	1		N/A	N/A
< 2500 g	27/103 (26.2)	18/90 (20.0)	1.42 (0.72–2.81)	0.31		
Gestational age categories						
≥ 38 weeks	65/100 (65.0)	27/89 (69.7)	1	_	N/A	N/A
< 38 weeks	35/100 (35.0)	62/89 (30.3)	1.24 (0.67–2.28)	0.497		
Sex						
Female	35/103 (34.0)	40/90 (44.4)	1	_	N/A	N/A
Male	68/103 (66.0)	50/90 (55.6)	1.55 (0.86–2.80)	0.138		
Apgar score at 5 min						
≥ 4	22/97 (22.7)	66/86 (76.7)	1	_	1	
0–3	75/97 (77.3)	20/86 (23.3)	11.2 (4.94–25.6)	< 0.001	0.94 (0.66–1.35)	0.752
Time to First Breath						
< 20 min	19/62 (30.6)	47/63 (74.6)	1	_	1	
≥ 20 min	43/62 (69.4)	16/63 (25.4)	6.65 (2.77–16.0)	< 0.001	0.48 (0.08–2.76)	0.413
Sarnat staging						
Stage 2 (moderate encephalopathy)	13/103 (12.6)	71/90 (78.9)	1		1	
Stage 3 (severe encephalopathy)	90/103 (97.4)	19/90 (21.1)	25.9 (9.17–73.0)	< 0.001	51.5 (5.86–453)	< 0.001
Thompson score						
≤ 10	3/65 (4.6)	40/83 (48.2)	1		1	
> 10	62/65 (95.4)	43/83 (51.8)	19.2 (4.69–78.8)	< 0.001	1.21 (0.12–11.9)	0.871
Arterial blood gas, pH						
≥ 7.00	11/92 (12.0)	36/71 (58.7)	1		1	
< 7.00	81/92 (88.0)	35/71 (49.3)	7.57 (3.18–18.0)	< 0.001	3.40 (0.45–25.5)	0.234
Arterial blood gas, base deficit (mmols/L)						
≤ 16	12/92 (13.0)	36/85 (42.4)	1		1	
> 16	80/92 (87.0)	49/85 (57.6)	4.90 (2.22–10.8)	< 0.001	0.59 (0.09–3.91)	0.586

Similar findings were found when comparing non-survivors to survivors with all neonates included irrespective of cooling status (entire cohort), severe encephalopathy was the only factor associated with mortality on multivariate analysis (OR 5.91, 95% CI 2.61–13.39; *p* < 0.001).

## Discussion

This study reports on outcomes at hospital discharge in neonates with moderate to severe encephalopathy over a 5-year period. The neonates were cooled according to standard hospital protocol that was derived from the TOBY criteria [22]. Overall, about two-thirds of neonates with moderate-to-severe encephalopathy total cohort were cooled, with this proportion of cooled neonates reducing from 74.6% in 2015 to 66.2% in 2017 with a further reduction to 61.2% in 2018. Amongst those who qualified for cooling and were not cooled the common reasons for not cooling were severity of clinical condition of the neonate, cooling equipment unavailability and late presentation at the treatment center. Overall mortality in neonates with moderate-to-severe encephalopathy was 29.0%, being 17.0% in cooled and 53.4% in the non-cooled group. On multivariate analysis, severe encephalopathy was the only factor found to be associated with mortality.

The proportion of neonates with moderate-to-severe encephalopathy who were cooled decreased over time most likely because at the initial phase of implementation of this intervention it was often noted that neonates with mild encephalopathy were cooled because of being misevaluated as having moderate-to-severe encephalopathy. This could also be explained by subsequent change in the Thompson score used in the inclusion criteria for cooling from a score of ≥ 7 to that of ≥ 10 at the beginning of 2018. This highlights the importance of appropriate selection of neonates who are cooled and ensuring that when outcomes are compared between different studies, the characteristics of neonates cooled in the different studies are clearly described.

Mortality rate of 17% in cooled neonates observed in this study is similar to rates of 13–20% reported in other observational studies from South Africa, from similar settings with access to mechanical ventilation or intensive care when needed [19, 20]. A mortality rate of 30% was reported in a study in Uganda, where access to intensive care was limited [18]. This highlights the importance of offering other supportive care that neonates with moderate-to-severe encephalopathy might need in addition to cooling, thus cooling must not be offered in isolation without other services that these infants might need. Therefore, outcomes of neonates who require cooling must be assessed with the background understanding of the healthcare system especially in LMIC where clinical services might vary widely, compared to HIC.

The sicker neonates as evidenced by lower 5-min Apgar score, metabolic acidosis, delayed onset of respiration and high Thompson score were more likely not to survive irrespective of whether these neonates were offered cooling or not, and this is consistent with many previous studies [23–25]. Lower 5-min Apgar scores of 0–3 have been reported to be associated with high mortality rates [23–25]. Metabolic acidosis evidenced by low pH and high base deficit have been reported to be clinical biomarkers that can predict mortality especially in the cooling era [24, 26, 27]. Thompson scores in non-cooled cohorts better predicted mortality than morbidity with scores between 10–14 reported to have higher mortality and an abnormal outcome which included cerebral palsy or neurodevelopmental impairment [4, 28, 29]. In a cohort of neonates who were cooled, Thompson score of ≥ 15 were associated with mortality and epilepsy, neurological and cognitive impairment [30].

The only factor found to be associated with mortality in the entire cohort using multivariate logistic regression analysis was severe encephalopathy. Studies from other LMICs, reported similar findings. A Tanzanian asphyxia cohort study reported an overall mortality of 9.1% with 84.7% occurring in the severe encephalopathy group [31]. In an 8-year retrospective review in Nigeria, the incidence was reported as 28/1 000 live births, with an overall mortality rate of 27.3%, with 52% mortality rate in those with severe asphyxia [32]. Though many studies from LMICs were not randomized clinical trials, they suggest that, it might be appropriate not to cool babies with severe encephalopathy especially in settings where resources are limited. Another South African study reported a high number of deaths in neonates with severe encephalopathy [20]. Sarnat scoring systems used in the NICHD-NRN cooling trial predicted mortality in 71% of cooled neonates [26]. A Sarnat score > 30 in neonates with severe encephalopathy were more likely to die despite hypothermia [24].

Therapeutic hypothermia trials in HIC report a reduction in mortality and favorable neurodevelopmental outcomes at 18–24 months with a number needed to treat of 7 [10]. Studies from LMICs are conversely steeped in controversy as to whether cooling is protective in these settings or not. With the results of the recent HELIX trial, the authors called for an immediate stop to cooling due to an increase in mortality in the neonates who were cooled. The authors also concluded that perhaps the lack of benefit of cooling is due to the population of neonates selected to manage with TH [12]. To the contrary, a meta-analysis by Abate et al. report that TH decreased mortality in HIC as well as LMICs, with the highest reduction of mortality seen in LMICs [11]. While this current study reports short-term outcomes of neonates who were cooled but it is important that long-term outcomes be assessed so that the relative low mortality rate at hospital discharge is not outweighed by high mortality post-discharge and increased risk for abnormal neurodevelopmental outcomes. In a separate study in our setting, we reported an abnormal outcome in 32% of neonates who were cooled when assessed at 18–24 months [15]. Another study from South Africa reported severe neurodevelopmental outcomes in 18% of infants assessed at 1 year of age [20].

Limitation of this retrospective observational study include the fact that there was incomplete information especially on the reasons for not cooling neonates who were considered to have moderate-to-severe encephalopathy and were not cooled. However, we do not believe that the missing data would have significantly affected the findings in this study. Another limitation is the lack of information on aEEG, cranial ultrasound and MRI as these could be used as biomarkers of severity of encephalopathy in assessing the characteristics of neonates who were cooled in this study.

## Conclusion

This study reports an overall mortality rate of 29% with a relatively low mortality rate (17%) in neonates who were managed with TH. There is however, a selection bias as only 10% of the neonates in the severe encephalopathy group were offered TH. In both cooled and non-cooled groups, severe encephalopathy (HIE 3) was associated with a higher risk of mortality. This suggests that in resource constraint settings it might be appropriate to be selective in who is offered cooling based on the severity of encephalopathy. Acknowledging that a large clinical trial from LMICs reported high mortality rate, it is critical that more prospective clinical trials are conducted in LMIC settings to assess whether cooling is beneficial or harmful in neonates with intrapartum asphyxia. Future studies should look at all variables that can improve in assessing the severity of brain damage and factors that might affect the overall outcomes of neonates with moderate-to-severe encephalopathy including availability of equipment, intensive care beds and other diagnoses that might negatively impact on survival including sepsis. Critical will be the follow-up of those that are cooled to assess the long-term neurological outcomes and further identify factors associated with good neurological outcomes amongst survivors.

## Data Availability

The datasets used and/or analyzed during the current study are available from the corresponding author on reasonable request.
